# Cloning, molecular evolution and functional characterization of ZmbHLH16, the maize ortholog of OsTIP2 (OsbHLH142)

**DOI:** 10.1242/bio.026393

**Published:** 2017-10-02

**Authors:** Yongming Liu, Jia Li, Gui Wei, Yonghao Sun, Yanli Lu, Hai Lan, Chuan Li, Suzhi Zhang, Moju Cao

**Affiliations:** 1 Maize Research Institute, Sichuan Agricultural University, 611130 Chengdu, China; 2 Key Laboratory of Biology and Genetic Improvement of Maize in Southwest Region, Ministry of Agriculture, 611130 Chengdu, China; 3 Tropical Crops Genetic Resources Institute, Chinese Academic of Tropical Agricultural Sciences, 571737 Danzhou, China; 4 National Key Lab of Crop Genetic Improvement, Huazhong Agricultural University, 430070 Wuhan, China

**Keywords:** Maize, bHLH transcription factor, Coexpression analysis, Molecular evolution, Male reproduction

## Abstract

The transcription factor ZmbHLH16, the maize ortholog of OsTIP2 (OsbHLH142), was isolated in the present study. Tissue expression analysis showed that ZmbHLH16 is preferentially expressed in male reproductive organs. Subcellular location analysis of ZmbHLH16 via rice protoplast indicated that it is located in the nucleus. Through nucleotide variation analysis, 36 polymorphic sites in ZmbHLH16, including 23 single nucleotide polymorphisms and 13 InDels, were detected among 78 maize inbred lines. Neutrality tests and linkage disequilibrium analysis showed that ZmbHLH16 experienced no significant evolutionary pressure. Yeast one-hybrid experiment showed that the first 80 residues in the N-terminus of ZmbHLH16 had transactivation activity, whereas the full length did not. Genome-wide coexpression analysis showed that 395 genes were coexpressed with ZmbHLH16. Among these genes, the transcription factor ZmbHLH51 had similar expression pattern and identical subcellular localization to those of ZmbHLH16. Subsequently, the interaction between ZmbHLH51 and ZmbHLH16 was verified by yeast two-hybrid experiment. Through yeast two-hybrid analysis of series truncated ZmbHLH16 fragments, we found not only the typical bHLH domain [175-221 amino acids (a.a.)], but also that the 81-160 a.a. and 241-365 a.a. of ZmbHLH16 could interact with ZmbHLH51. All these results lay the foundation for further understanding the functions of ZmbHLH16.

## Abstract (precis)

**Summary:** ZmbHLH16, the maize ortholog of OsTIP2 (OsbHLH142), its subcellular location, molecular evolution, transcriptional activity domains and heterodimerization domains with ZmbHLH51 are analyzed.

## INTRODUCTION

Maize is the most important crop in the world for its utilization in food and industrial materials. At present, there is a rising demand for maize crop yields (Ray et al., 2013). Benefitting from hybrid vigor, male sterility can be used for hybrid maize seed production to increase crop yield and improve food security (Zhang and Liang, 2016). Therefore, the study of male sterility is of great value for application. Until now, several maize genic male sterile (GMS) genes, such as *ms8* (Wang et al., 2013), *ms9* (Albertsen et al., 2016), *ms26* (Djukanovic et al., 2013), *ms32* (Moon et al., 2013), *ms44* (Fox et al., 2017) and ms*45* (Albertsen et al., 1993), have been cloned and illuminated for their abortion mechanism. These findings not only contribute to maize heterosis utilization but also expand our understanding of maize male reproduction. Conventionally, GMS genes are mainly identified through mutant analysis. With the development of gene-editing technology, more male sterile genes are now known from the direct editing of some key genes involving pollen development in maize (Mark Cigan et al., 2017; Qi et al., 2016; Svitashev et al., 2016; Svitashev et al., 2015). As a result, the identification of key genes in male reproduction is becoming increasingly important. More than 10,000 genes have been shown to be expressed specifically in maize male fertility development (Ma et al., 2008). Transcription factors (TFs) play key roles in regulating their spatial- and temporal-specific expression. Interestingly, TFs might also be the target genes of some small RNAs in plant meiotic processes (Alonsoperal et al., 2010; Chen, 2004; Yu et al., 2013). These above findings indicate that TFs play important roles in plant reproduction. In maize, a total of 2298 TFs have been identified, and some show tissue-specific expression (Jiang et al., 2012). Key TFs in maize meiosis have been identified using high-throughput techniques such as microarray hybridization and transcriptome sequencing (Dukowic-Schulze et al., 2014a,b; Zhang et al., 2014). However, only two pollen development-related transcription factors, *ms9* (R2R3-MYB) and *ms32* (bHLH), have been cloned in maize (Albertsen et al., 2016; Moon et al., 2013). To reveal the regulatory mechanism of maize pollen formation, it is imperative to identify additional TFs involved in maize male fertility.

The basic helix-loop-helix (bHLH) proteins compose one of the largest plant transcription factor families. In rice and maize, 178 and 276 bHLH TFs have been identified, respectively (Carretero-Paulet et al., 2010; Jiang et al., 2012; Li et al., 2006a). Abnormal functions of some bHLH TFs may lead to plant male sterility. In *Arabidopsis*, 10 bHLH proteins related to pollen development have been isolated: AtAMS (Sorensen et al., 2003; Xu et al., 2014), AtDYT1 (Feng et al., 2012; Zhang et al., 2006), AtbHLH10 (Zhu et al., 2015a), AtbHLH89 (Zhu et al., 2015a), AtbHLH91 (Zhu et al., 2015a), AtJAM1 (Nakata et al., 2013), AtJAM2 (Nakata and Ohme-Takagi, 2013), AtJAM3 (Nakata and Ohme-Takagi, 2013), AtMYC5 (Figueroa and Browse, 2015) and AtBIM1 (Xing et al., 2013). Moreover, these male sterile mutants have unique male reproduction-deficient characteristics. For example, the *ams* mutant exhibits total male sterility without any visible pollen; the *dyt1* mutant can produce few pollen grains with a low rate of self-fertility; and the single mutants of *AtbHLH10*, *AtbHLH89* and *AtbHLH91* develop normally, with only their various double and triple combinations defective in pollen development (Li et al., 2006b; Sorensen et al., 2003; Zhu et al., 2015a). These differences in male sterile characteristics might result from the functional divergence of bHLH TFs. Tapetal cells provide energy and nutrition for microspore development via programmed cell death (PCD) at appropriate anther developmental stages (Zhang et al., 2008a). In rice, bHLH TFs including OsUDT1 (Jung et al., 2005), OsTDR (Li et al., 2006b; Zhang et al., 2008b), OsEAT1/OsDTD1 (Ji et al., 2013; Niu et al., 2013) and OsTIP2 (OsbHLH142) (Fu et al., 2014; Ko et al., 2014) have been found to be essential for anther tapetal cell development. These above rice bHLH TF dysfunctions lead tapetal cells to undergo abnormal PCD, thereby causing complete male sterility. In conclusion, all of the above show that bHLH TFs play important roles in regulating stamen development. At present, few studies exist on bHLH TFs related to maize male fertility, and there is a need to characterize more bHLH proteins in maize male reproduction.

*TIP2* (OsbHLH142) acts as a key regulator of tapetum development in rice (Fu et al., 2014; Ko et al., 2014). The *tip2* mutant displays complete male sterility, with three undifferentiated anther wall layers (epidermal, fibrous and middle layer) and abortion of tapetal programmed cell death (Fu et al., 2014). In this study, the transcription factor ZmbHLH16, which is homologous to OsTIP2 (OsbHLH142), was isolated from maize. To determine ZmbHLH16 molecular function, its structure, phylogeny, expression and subcellular localization, molecular evolution and regulatory characteristics were investigated.

## RESULTS

### ZmbHLH16 is a typical bHLH transcription factor

**Fig. 1.**
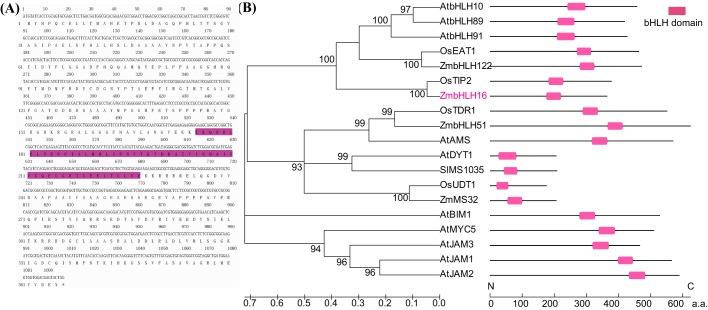
**Structure and phylogenetic analysis of ZmbHLH16.** (A) Nucleotide and deduced amino acid sequences of ZmbHLH16 CDS. Shaded regions are the conserved DNA-binding domains of the bHLH protein. Highlighted letters show conserved tryptophan residues in the bHLH domain. (B) Phylogenetic relationship of ZmbHLH16 and other bHLH proteins related to microspore development. The two scale bars indicate branch length and amino acid length.

The ZmbHLH16 coding sequence (CDS) was obtained from the maize inbred line A619. Further analysis showed that the ZmbHLH16 CDS contained 1098 bp encoding a protein of 365 amino acids (a.a.). The ZmbHLH16 protein had 66.85% a.a. sequence identity with OsTIP2 (OsbHLH142). Analysis with NCBI CD software revealed that the a.a. sequence of ZmbHLH16 included a typical bHLH DNA-binding domain (Fig. 1A). A previous study showed that the bHLH interaction and function (BIF) domain participates in bHLH protein localization, transcriptional activity and dimerization (Cui et al., 2016). The BIF domain was also found in the C-terminal (288-363 a.a.) region of ZmbHLH16 in our study. Furthermore, phylogenetic analysis of 17 bHLH transcription factors related to microspore development illustrated that these bHLH TFs were highly conserved, with most bootstrap values >90%. TIP2, TDR and EAT1 are key regulators involved in rice anther development, and the gene models GRMZM2G021276 (ZmbHLH16), GRMZM2G139372 (ZmbHLH51) and AC233960.1_FG005 (ZmbHLH122) had the highest homology scores with them, respectively (Fig. 1B). Together, the above results indicated that ZmbHLH16 protein possessed the typical conserved domain of bHLH TFs and might play a crucial role in male reproduction in maize.

### ZmbHLH16 is highly evolutionarily conserved in maize germplasm

To analyze its molecular evolution, the DNA sequences of ZmbHLH16 were amplified from 78 maize inbred lines. The ZmbHLH16 genomic sequence is divided into seven regions (Table S1). Based on the minor allele frequency (MAF) ≥0.05, 36 polymorphism sites within ZmbHLH16 were identified, including 23 single nucleotide polymorphisms (SNPs) and 13 InDels, with one SNP/InDel per 109/193 bp (Table S1). Among 23 SNPs, 13 (57%) and 10 (43%) involved transitions and transversions, respectively. Further analysis showed that there were four a.a. variations in ZmbHLH16 among 78 inbred lines, with three mutations in the first exon and the fourth mutation in the second exon. Comparison analysis showed that nucleotide variations were not evenly distributed in ZmbHLH16, and introns had higher sequence diversity (3.1 polymorphisms/100 bp) than UTRs (1.24 polymorphisms/100 bp) and exons (1.18 polymorphisms/100 bp). Moreover, a nucleotide polymorphism test in DnaSP showed the highest nucleotide diversity ratio (π=6.15×10^−3^) in the first intron, but no significant nucleotide variation in the third intron or 3′-UTR (Table 1).

**Table 1.**
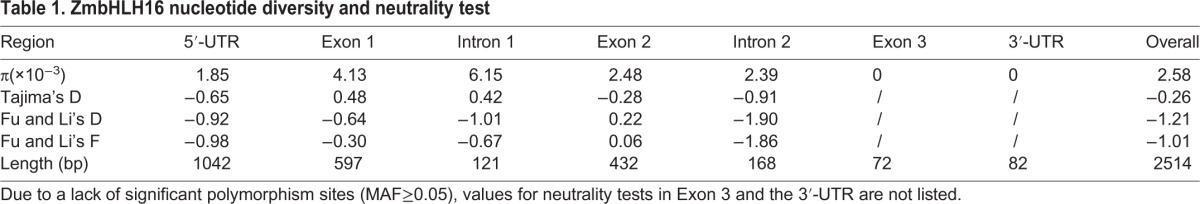
**ZmbHLH16 nucleotide diversity and neutrality test**

**Fig. 2.**
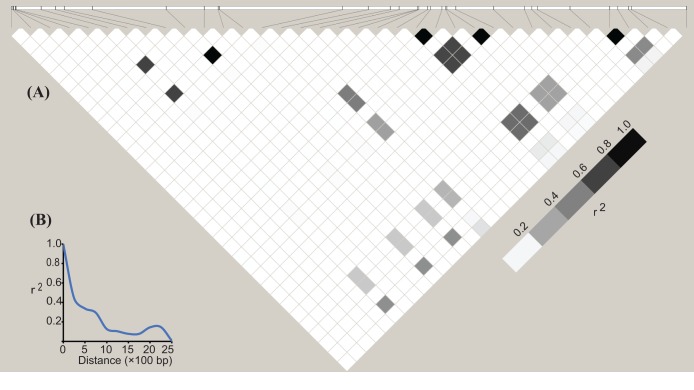
**LD block and LD decay pattern of ZmbHLH16.** (A) Matrix of pairwise LD of DNA polymorphisms (MAF≥0.05) in ZmbHLH16. The shaded boxes indicate the LD standard (*r*^2^). (B) LD decay in the DNA sequence of ZmbHLH16 in maize. The *x*-axis represents the distance between polymorphic sites, and the *y*-axis represents the average *r^2^* value for each distance category (250 bp).

To examine whether ZmbHLH16 has experienced selection pressure, various regions of ZmbHLH16 were assessed (Table 1). In Tajima's D test and Fu and Li's test, no significant difference was obtained across all regions of ZmbHLH16. This result indicated that ZmbHLH16 experienced no significant selective pressure and underwent neutral selection. Elevated linkage disequilibrium (LD) is usually expected for genes under selection (Bomblies and Doebley, 2005). Thus, to further confirm whether ZmbHLH16 underwent directional selection, its LD patterns and LD decay were also calculated. In the LD matrix, no obvious LD block was found in the ZmbHLH16 genome sequence (Fig. 2A). A schematic diagram of LD decay represented by plots of *r*^2^ showed that the LD level dropped to 0.1 at ∼1300 bp (Fig. 2B), indicating a more rapid decay rate than the average 1.5 kb of several genes under selection in maize (Remington et al., 2001). Therefore, our above results also reflected the conserved evolution of ZmbHLH16 in the maize germplasm.

### Only the N-terminal first 80 residues of ZmbHLH16 have transactivation activity

**Fig. 3.**
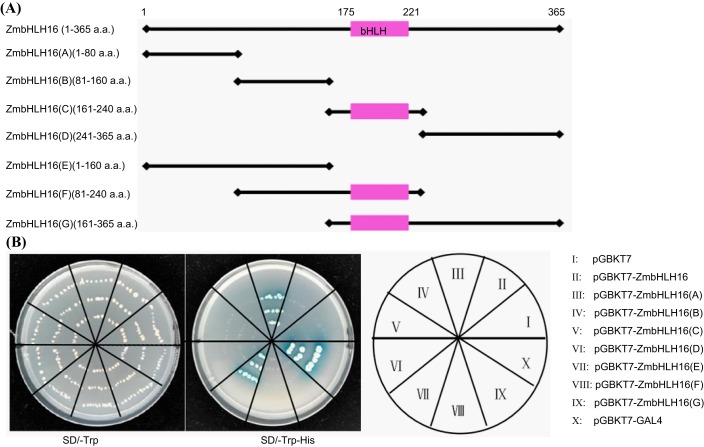
**Transactivation activity assays of ZmbHLH16 in yeast.** (A) Diagram of ZmbHLH16 activation domain. (B) Growth of yeast containing various fragments of ZmbHLH16 on selective media (SD/-Trp and SD/-Trp-His).

To identify the activating function of ZmbHLH16, eight fragments of ZmbHLH16 were analyzed in yeast (Fig. 3A). Yeast cells with pGBKT7-ZmbHLH16 (A) (1-80 a.a.) or pGBKT7-ZmbHLH16 (E) (1-160 a.a.) grew normally on both synthetic dropout (SD)/-Trp and SD/-His-Trp selective media and turned the indicator blue. However, the other six yeast transformants could only live on the SD/-Trp medium (Fig. 3B). Based on the growth conditions of the transformants containing ZmbHLH16 (A) (1-80 a.a.), (B) (81-160 a.a.) and (E) (1-160 a.a.), it was not difficult to find that the N-terminal first 80 a.a. of ZmbHLH16 possessed transcriptional activation activity. According to the yeast growth difference between the whole coding region (1-365 a.a.) and region (E) (1-160 a.a.), it was inferred that domain (G) (161-365 a.a.) of ZmbHLH16 might inhibit its transcriptional activation activity. In conclusion, the above results indicated that the first 80 a.a. in the N-terminus of bHLH16 could activate transcription in yeast, whereas the full-length version did not.

### ZmbHLH16 coexpresses with many male reproduction-related genes

**Fig. 4.**
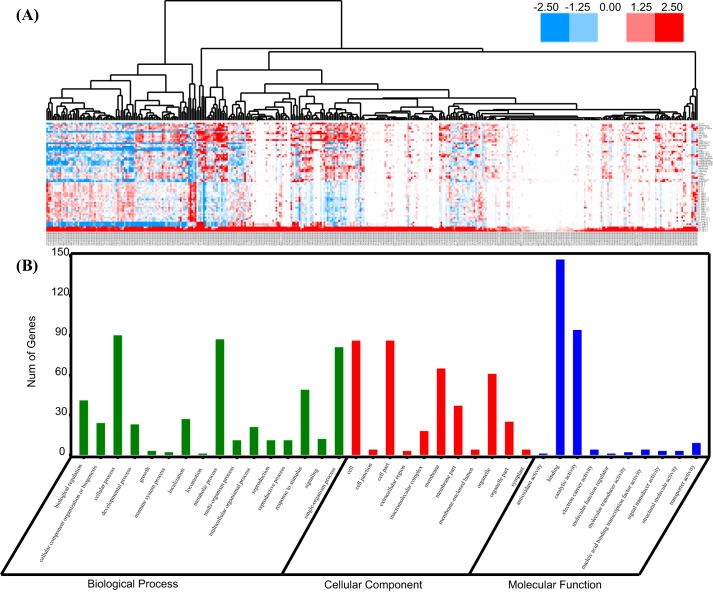
**Expression patterns and GO annotations of ZmbHLH16 coexpressed genes.** (A) A cluster of 395 coexpressed genes based on expression characteristics. Gene expression data were downloaded from q-teller (http://www.qteller.com/qteller4/); the bar indicates the relative gene expression level, which was log_2_-normalized (original data+1). (B) GO analysis of 395 coexpressed genes.

Functionally associated genes are more likely to share similar expression patterns (Fu and Xue, 2010). Coexpression analysis was therefore conducted to identify potential ZmbHLH16 cooperators. A total of 395 maize genes were coexpressed with ZmbHLH16, the Pearson's correlation coefficient (PCC) values of which were >0.6 (Fig. 4A; Table S2). Among them, there were three male sterile genes, including *ms8* (GRMZM2G119265), *ms26* (GRMZM2G091822) and *ms44* (AC225127.3_FG003), which shared expression PCC values of 0.9937, 0.8375 and 0.9964 with ZmbHLH16, respectively. Through searching in the PMRD database, among 395 coexpression genes, 34 homologous genes had been annotated as involved in *Arabidopsis* male reproduction (Table S6). The similar expression pattern to a number of plant reproduction-related genes indicated that ZmbHLH16 might be closely associated with maize male fertility.

Next, the 395 coexpressed genes were subjected to Gene Ontology (GO) term analysis (Fig. 4B; Table S2). In the cellular component, 155 GO terms were enriched and most of these genes were categorized under cells, cell parts, membranes and organelles. For the molecular function category, binding and catalytic activity were the most abundant subcategories. Similarly, previous reports have confirmed that the binding activity and catalytic activity functions are essential for alterations in male fertility (Mei et al., 2016; Qu et al., 2015; Zhu et al., 2015b). For biological processes, there were 780 enriched GO terms, cellular processes and metabolic processes, and single-organism processes were the most abundant clusters. Through hypergeometric test at the 0.05 significance level, it was found that some enriched GO terms, such as pollen wall assembly, pollen exine formation, pollen development and gametophyte development, reached significant levels compared with the maize background (Table S3). These results supported that ZmbHLH16 might participate in maize pollen formation. Moreover, in the reproduction GO term (GO:0000003), a bHLH transcription factor family member, ZmbHLH51 (GRMZM2G139372), was found, which shared a PCC score of 0.8990 with ZmbHLH16. ZmbHLH51 was homologous to the male sterile gene OsTDR. Accordingly, ZmbHLH51 might be an important factor in maize pollen development. Some studies have indicated that the interactions among bHLH TFs are important for pollen development (Niu et al., 2013; Zhu et al., 2015a). Therefore, we next aimed to analyze the interaction between ZmbHLH16 and ZmbHLH51.

### ZmbHLH16 and ZmbHLH51 have similar expression characteristics

**Fig. 5.**
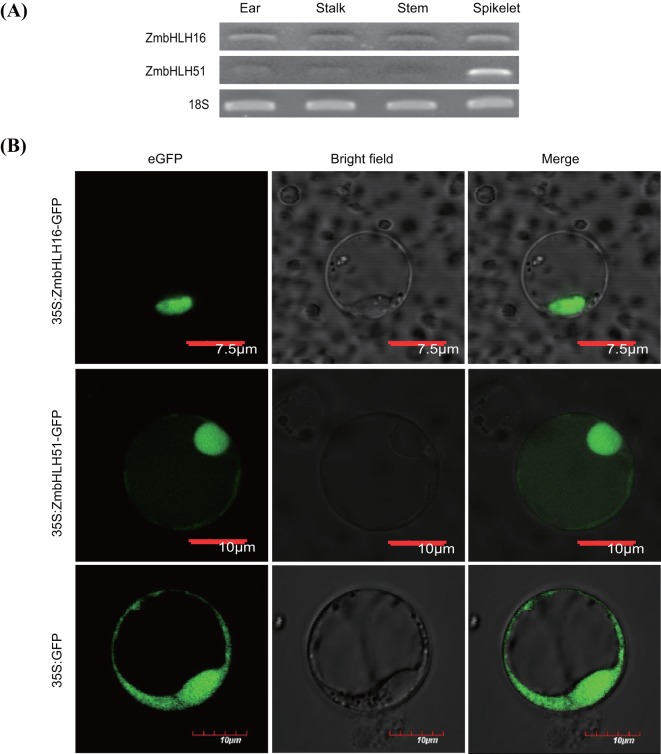
**Expression characteristics of ZmbHLH16 and ZmbHLH51.** (A) Semi-quantitative analysis of ZmbHLH16 and ZmbHLH51 in various organs. The expression of 18S was taken as the reference. (B) Subcellular localization analysis of ZmbHLH16 and ZmbHLH51 in rice protoplasts.

The expression patterns of ZmbHLH16 and ZmbHLH51 were simultaneously analyzed using semi-quantitative polymerase chain reaction (PCR) for reproductive and vegetative organs. Both ZmbHLH16 and ZmbHLH51 showed a higher expression level in spikelets than other organs (Fig. 5A). This finding indicated that ZmbHLH16 and ZmbHLH51 might be closely associated with maize male fertility.

Based on the above results, ZmbHLH51 is homologous to the male sterile gene *OsTDR* and might interact with ZmbHLH16. Therefore, the subcellular localizations of ZmbHLH16 and ZmbHLH51 were both analyzed in rice protoplast. As depicted in Fig. 5B, the recombinant fusion proteins ZmbHLH16-enhanced Green Fluorescent Protein (eGFP) and ZmbHLH51-eGFP were both located in the nucleus only, whereas the control eGFP was localized to both the cytoplasm and the nucleus. The similar expression profiles and protein localization patterns between ZmbHLH16 and ZmbHLH51 suggested they might function correlatedly.

### ZmbHLH51 interacts with ZmbHLH16

**Fig. 6.**
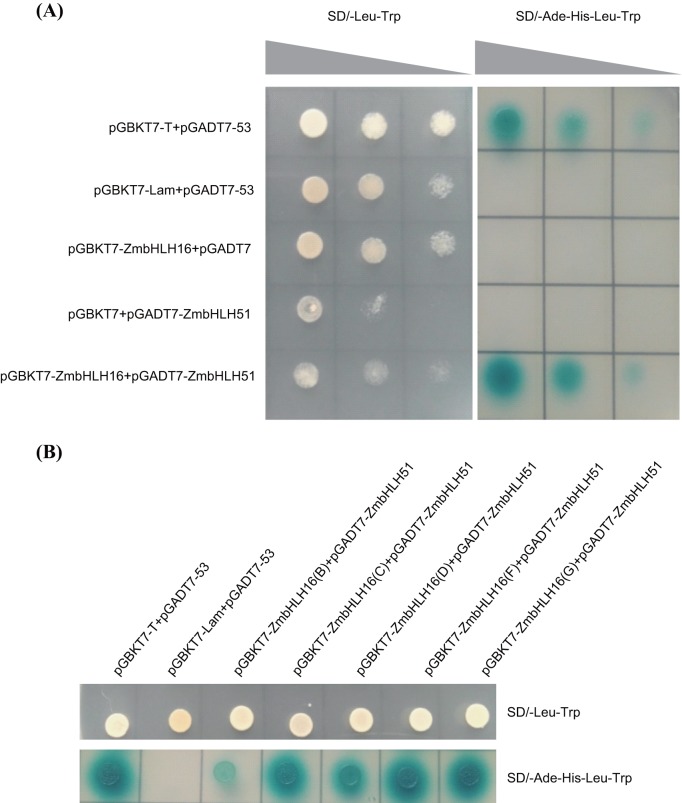
**Determination of the interaction between ZmbHLH16 and ZmbHLH51 using Y2H analysis.** (A) Y2H analysis of the interaction between ZmbHLH16 and ZmbHLH51 proteins. Each transformant was stained in media with three relative concentrations (1, 0.1, 0.01) from left to right. (B) Y2H mapping of domains involved in the ZmbHLH16-ZmbHLH51 interaction. Regions without transcriptional activation activity, including ZmbHLH16 (B) (81-160 a.a.), (C) (161-240 a.a.), (D) (241-365 a.a.), (F) (81-240 a.a.) and (G) (161-365 a.a.), were chosen for analysis.

Because the aforementioned results indicated that the two bHLH TFs ZmbHLH16 and ZmbHLH51 had similar expression characteristics and subcellular localization patterns, a yeast two-hybrid (Y2H) assay was used to verify the interaction between ZmbHLH16 and ZmbHLH51. As shown in Fig. 6A, those yeast cells merely containing pGBKT7-ZmbHLH16 or pGATD7-ZmbHLH51 could only live on the SD/-Leu-Trp but not SD/-Ade-His-Leu-Trp. In comparison, those yeast cells containing both pGBKT7-ZmbHLH16 and pGADT7-ZmbHLH51 were able to grow on both SD/-Leu-Trp and SD/-Ade-His-Leu-Trp media, similar to the positive control. These results proved that the interaction between ZmbHLH16 and ZmbHLH51 really existed.

To map the domains involved in the ZmbHLH16-ZmbHLH51 interaction, fragments without transcriptional activation activity, including ZmbHLH16 (B) (81-160 a.a.), (C) (161-240 a.a.), (D) (241-365 a.a.), (F) (81-240 a.a.) and (G) (161-365 a.a.), were further analyzed using Y2H assays. The conserved bHLH domain is reported to participate in protein homo- or heterodimerization (Pires and Dolan, 2010). As expected, the regions containing the bHLH domain, i.e. (C) (161-240 a.a.), (F) (81-240 a.a.) and (G) (161-365 a.a.), could grow normally on SD/-Ade-His-Leu-Trp media and turned the media blue (Fig. 6B). Interestingly, transformants (B) and (D), which lacked the bHLH domain, also survived on the SD/-Ade-His-Leu-Trp synthetic dropout medium. These results manifested that not only the bHLH domain but also other regions in ZmbHLH16 were sufficient and necessary for its heterodimerization with ZmbHLH51.

## DISCUSSION

Male reproduction is a complicated process in plants that involves thousands of genes and many biological processes (Dukowic-Schulze and Chen, 2014; Rutley and Twell, 2015; Zhou and Pawlowski, 2014). Several genes and regulatory networks involved in plant male reproductive development are found to be conserved, particularly in pollen wall development between *Arabidopsis* and rice (Gómez et al., 2015; Shi et al., 2015; Zhang et al., 2016). This phenomenon provides the possibility of elucidating key genes in other species based on homology analysis. Thus, here, we isolated ZmbHLH16 based on homology cloning from OsTIP2, which has been reported to be a master regulator of pollen formation.

In this study, the molecular evolution of ZmbHLH16 was investigated. In the analysis of selective pressure, no significant signal was found in ZmbHLH16 according to Tajima's D and Fu and Li's tests. Moreover, a lower nucleotide diversity ratio (π=2.58×10^−3^) was observed in all regions of ZmbHLH16 than in the average (π=6.3×10^−3^) of 18 maize genes in previous reports (Ching et al., 2002). This finding implied weak or no natural selection pressure on ZmbHLH16 and provided evidence that ZmbHLH16 is highly evolutionarily conserved in maize. The target gene sequence polymorphism also reflects evolutionary pressure during maize improvement (Wang et al., 2005). Previous studies found one polymorphic site per 60.8 bp in maize (Ching et al., 2002). In the present experiments, a lower frequency was obtained for ZmbHLH16 in 78 maize inbred lines (one SNP or InDel every 69.8 bp). The global LD decay of ZmbHLH16 investigated in our study (*r^2^*<0.1 within 1300 bp) was also less than the average intragenic level (*r^2^*<0.1 within 1500 bp) (Remington et al., 2001). The above nucleotide polymorphism testing results confirmed the conserved evolution of ZmbHLH16. The conserved molecular evolution of ZmbHLH16 further hinted at its crucial function in maize male reproduction.

Most bHLH proteins consist of a classical helix-loop-helix (HLH) domain to form homo- or heterodimers with other HLH proteins to regulate downstream target genes (Murre et al., 1989). bHLH-bHLH or bHLH-MYB complexes have been reported to be involved in plant fertility (Chen et al., 2016; Niu et al., 2013; Qi et al., 2015). Our experiments showed that ZmbHLH16 lacks the ability of transcriptional activation. Thus, we speculate that ZmbHLH16 might regulate target gene expression by interacting with other proteins. One of its interacting factors, ZmbHLH51, was identified and confirmed using genome-wide coexpression and Y2H analyses. In rice, the BIF domain is necessary for DYT1-bHLH protein dimerization (Cui et al., 2016). The present study showed that the BIF domain is also present in ZmbHLH16 (D) (241-365 a.a.) and participates in the interaction between ZmbHLH16 and ZmbHLH51. Interestingly, we noticed that not only the conserved bHLH and BIF domains but also the ZmbHLH16 (B) (81-160 a.a.) region could form heterodimers with ZmbHLH51. Moreover, the ZmbHLH16 (G) (161-365 a.a.) fragment may have a negative effect on activation, leading to a reduced transcription activation capacity of the full-length ZmbHLH16 protein. Taken together, our findings provide new evidence that in bHLH proteins, other regions are of importance for their molecular function in addition to the typical bHLH and BIF domains.

The normal tapetal cells specification were regulated by many factors and its abnormal development might cause dysfunctional microspore (Zhang and Yang, 2014). It was recently reported that ZmbHLH16 was a candidate gene for the maize ms23 mutant (Nan et al., 2017). The tapetal layer of the ms23 mutant undergoes abnormal periclinal division instead of tapetal differentiation (Chaubal et al., 2000). In Nan et al., (2017), the researchers mainly focused on the abortion mechanism in the ms23 mutant, combining RNA-seq with proteomics data. These authors also detected the interaction between ZmbHLH16 and ZmbHLH51. In contrast, we paid more attention to the ZmbHLH16 nucleotide polymorphisms, molecular evolution, expression features, subcellular location and regulatory mechanisms. Through coexpression analysis, a group of genes potentially involved in maize male reproduction were also revealed in this study. Our results might help uncover the mechanism of ZmbHLH16 regulating the pollen abortion in the ms23 mutant.

## MATERIALS AND METHODS

### Plant materials

Spikelets from maize inbred line A619 were collected for ZmbHLH16 (GRMZM2G021276_T02) and ZmbHLH51 (GRMZM2G139372_T07) CDS cloning. Ears, main stalks, stems and spikelets were taken from maize inbred A619 for ZmbHLH16 expression analysis. Seeds from 78 inbred lines (Table S4) were used to amplify the genome sequence of ZmbHLH16.

### DNA and RNA extraction

Genomic DNA was extracted from seeds using a modified cetyltrimethylammonium bromide (CTAB) method (Porebski et al., 1997). Total RNAs were isolated from the above frozen samples with TRIzol reagent (Takara, Beijing, China) and DNase I to eliminate any genomic DNA. One microgram of total RNA from each sample was used to synthesize cDNA via the PrimeScript™ RT Reagent Kit (Takara).

### CDS cloning of ZmbHLH16 and phylogenetic analysis

BlastP (https://blast.ncbi.nlm.nih.gov/) was used to identify male fertility related bHLH homologous genes in the maize genome. The CDS of ZmbHLH16 was amplified from cDNA templates of A619 spikelets with the following primers: 5′-ATGTATCACCCGCAGTGCGAGCT-3′ and 5′-TGTACTCGTCCACCACTTCCAT-3′. High-fidelity KOD FX (Toyobo, Osaka, Japan) was used for gene cloning according to the manufacturer's instructions. The purified PCR products were inserted into the pEASY Blunt Simple cloning vector (TransGen, Beijing, China) and sequenced by Tsingke Biotech with an ABI 3730XL DNA Analyzer. The ZmbHLH16 a.a. sequence was acquired based on amplifying its CDS from A619 using the online program SoftBerry FGENESH (linux1.softberry.com/berry.phtml?topic=fgenesh&group=programs&subgroup=gfind), and its conserved domain was predicted using the NCBI CD tool (http://www.ncbi.nlm.nih.gov/Structure/cdd/wrpsb.cgi). Then, 16 bHLH TFs that were reported to be involved in microspore development were retrieved from Gramene (http://www.gramene.org/) to construct a phylogenetic tree using the neighbor-joining method with MEGA v5.10 (Kumar et al., 2008), and the robustness of the findings was verified via 1000 bootstrap replicates. The accession numbers of the 16 TFs are as follows: AtAMS (AT2G16910), AtDYT1 (AT4G21330), AtbHLH10 (AT2G31220), AtbHLH89 (AT1G06170), AtbHLH91 (AT2G31210), AtJAM1 (AT2G46510), AtJAM2 (AT1G01260), AtJAM3 (AT4G16430), AtMYC5 (AT5G46830), AtBIM1 (AT5G08130), OsUDT1 (OS07G0549600), OsTDR1 (OS02G0120500), OsEAT1 (OS04G0599300), OsTIP2 (OS01G0293100), ZmMS32 (GRMZM2G163233) and SlMS1035 (Solyc02g079810).

### Molecular evolution analysis of ZmbHLH16

The genomic sequences of ZmbHLH16, including its 5′ and 3′ untranslated regions (UTRs), were amplified from 78 maize inbred lines (see Table S4 for details) with the primers 5′-GGAAGGAGGAAACCAAGTCG-3′ and 5′-TGTAACGAGCAAGCGGATTTA-3′. PCR was performed according to the manufacturer's protocol using high-fidelity polymerase KOD FX (Toyobo). PCR-amplifying fragments were purified and sequenced directly using an ABI 3730XL DNA Analyzer manufactured by Tsingke Biotech. After ambiguous sequences were manually deleted, the sequence polymorphisms of ZmbHLH16 among the 78 maize inbred lines were analyzed using CodonCode Aligner 6.0.2 software (CodonCode Corporation, Dedham, MA, USA). For molecular evolution analysis, certain parameters were calculated as follows: (1) the nucleotide diversity of common pairwise nucleotide difference per site (π) with DnaSP 5.0 (Librado and Rozas, 2009); (2) in neutrality tests, the evolutionary pressure in ZmbHLH16 via Tajima's D test (Tajima, 1989) and Fu and Li's statistics (Fu and Li, 1993); (3) the LD matrix of ZmbHLH16 was characterized by evaluating *r*^2^ values based on SNPs and InDels (MAF≥0.05) in TASSEL 2.0 (Bradbury et al., 2007). An LD plot was obtained in Haploview 4.2 (Barrett et al., 2005), and the LD decay was assessed by averaging *r*^2^ values with a distance of 250 bp.

### Transactivation activity analysis of truncated ZmbHLH16

The ZmbHLH16 CDS contains 1098 bp encoding a protein with 365 a.a. To investigate its transcriptional activating ability and retain its conserved bHLH domain, the ZmbHLH16 CDS sequence was divided into four parts: the first three parts each contained 240 bp [labeled A (1-80 a.a.), B (81-160 a.a.) and C (161-240 a.a.)], and the last part contained 375 bp [labeled D (241-365 a.a.)] (Fig. 3A). At the same time, three other fragments, ZmbHLH16 (E) (1-160 a.a.), (F) (81-240 a.a.) and (G) (161-365 a.a.), were constructed, which overlapped the above four neighboring parts. The above seven parts, termed ZmbHLH16 (A)-(G), were artificially synthesized, and sequencing-confirmed. Next, eight fragments, including ZmbHLH16 CDS and ZmbHLH16 (A)-(G), were individually inserted into the pGBKT7 vector using the In-Fusion cloning method (Vazyme ClonExpress II One Step Cloning Kit, Vazyme Biotech, Nanjing, China) (see Table S5 for all primers used in the experiment). All recombinant pGBKT7 vectors were transformed into AH109 yeast strains (Tiandz, Beijing, China) via the lithium acetate-mediated approach. The transformants were cultivated on SD/-Trp medium for 2-3 days at 28°C. Bacterial PCR was used to identify positive clones. The positive clones were further cultured on SD/-His-Trp medium containing 50 mg l^−1^ χ-α-gal (Coolaber, Beijing, China) for 2-4 days at 28°C to test their transactivation activity. The pGBKT7 and pGBKT7-GAL4 AD vectors were used as negative and positive controls, respectively.

### Coexpression analysis of ZmbHLH16

For coexpression analysis, expression data of genome-wide maize genes in 20 tissues and 66 stages were obtained from q-teller (www.qteller.com/qteller4/), and the PCC values between ZmbHLH16 and other genes were calculated. Cluster3.0 (de Hoon et al., 2004) was used for target gene (PCC>0.6) cluster analysis based on Euclidean distance and complete linkage. A heatmap was drawn using Java Treeview (Saldanha, 2004). Next, to gain deeper insight into the molecular mechanism underlying ZmbHLH16, all target genes (PCC>0.6) were queried with E-value<1e^−5^ in the Plant Male Reproduction Database (http://202.120.45.92/addb/), which contains 548 male fertility-related genes in *Arabidopsis*. All maize gene sequences were retrieved from MaizeGDB (ftp://ftp.ensemblgenomes.org/pub/plants/release-29/fasta/zea_mays). To characterize the putative function of ZmbHLH16-coexpressed genes, GO terms for all target genes (PCC>0.6) were taken from AGRIgo (http://bioinfo.cau.edu.cn/agriGO/analysis.php), and GO enrichment analysis was performed using OmicShare tools (http://www.omicshare.com/tools).

### Expression characteristics of ZmbHLH16 and ZmbHLH51

Semi-quantitative expression analyses of ZmbHLH16 and ZmbHLH51 were conducted in vegetative and reproductive organs. Specific primer pairs including 5′-CCTCATGCACCTCATACC-3′ and 5′-CAGCTCCTGGATGTACTC-3′, 5′-CTGGAGGTCACCAACGTCAA-3′ and 5′-AGCGAGTCCCTCAGTCTGTC-3′ were designed for ZmbHLH16 and ZmbHLH51 expression analyses, respectively. The 18S gene was used as the internal control in this experiment, and its amplifying primers were 5′-CTGAGAAACGGCTACCACA-3′ and 5′-CCCAAGGTCCAACTACGAG-3′ (Hu et al., 2011).

The localization patterns of the ZmbHLH16 and ZmbHLH51 proteins were investigated through transient transformation in rice protoplasts. For ZmbHLH51 CDS cloning, the cDNA sequence was amplified with the primers 5′-GAGCAGTGATGTGAATTGCG-3′ and 5′-TCAAGCGAGGTATTGGAGGA-3′ using high-fidelity KOD FX polymerase from A619 and inserted it into the pEASY blunt-cloning vector. The CDSs of ZmbHLH16 and ZmbHLH51 lacking the stop codons were individually fused to the N-terminus of eGFP in pCAMBIA2300-P_35S_ by subcloning using the In-Fusion cloning method. Two recombinants, pCAMBIA2300-P*_35S_*:ZmbHLH16-eGFP and pCAMBIA2300-P*_35S_*:ZmbHLH51-eGFP, were constructed to assess the localization of these proteins. The empty pCAMBIA2300-P_35S_-eGFP vector was used as a control in this experiment. The recombinant vectors pCAMBIA2300-P*_35S_*:ZmbHLH16-eGFP and pCAMBIA2300-P*_35S_*:ZmbHLH51-eGFP, along with the control vector, were respectively transformed into rice protoplasts using polyethylene glycol (PEG), as described previously (Bart et al., 2006). The green signals (Ex=488 nm, Em=507 nm) were detected using a TCS-SP8 fluorescence microscope (Leica, Wetzlar, Germany).

### Protein–protein interactions

To confirm the interaction between ZmbHLH16 and ZmbHLH51, a Y2H assay was conducted. The CDSs of ZmbHLH16 and ZmbHLH51 were inserted into the pGBKT7 and pGADT7 vectors, respectively. The pGBKT7-ZmbHLH16 vector without autoactivation activity was constructed as above. The pGADT7-ZmbHLH51 vector was constructed using the In-Fusion cloning method by subcloning from pCAMBIA2300-P*_35S_*:ZmbHLH51. The recombinant vectors pGBKT7-ZmbHLH16 and pGADT7-ZmbHLH51 were co-transformed into AH109 yeast competent cells according to operating instructions. The transformants were cultivated on SD/-Leu-Trp medium at 28°C for 2-3 days, and positive clones were confirmed using PCR. Positive clones were further cultured on SD/-Ade-His-Leu-Trp medium containing 50 mg l^−1^ χ-α-gal at 28°C for 2-3 days. The vectors pGBKT7-T and pGBKT7-Lam were used as positive and negative controls, respectively. To confirm the interaction domain in ZmbHLH16, regions of ZmbHLH16 without autoactivation activity were inserted into the bait vector pGBKT7 and then co-transformed with the prey vector pGADT7-ZmbHLH51 into the AH109 yeast competent cells.

## Supplementary Material

Supplementary information
