# Supplementary material for: Cloning, molecular evolution and functional characterization of ZmbHLH16, the maize ortholog of OsTIP2 (OsbHLH142)
Source: Biol Open. 2017 Oct 2;6(11):1654–63. doi: 10.1242/bio.026393 (PMC5703606; doi:10.1242/bio.026393)
Supplement: Supplementary information [file biolopen-6-026393-s1.pdf]

Table S1. Single nucleotide polymorphisms (SNPs) and Insertion-deletion polymorphisms(InDels) of ZmbHLH16 among the 78 maize inbreds. Underlined letters represents the minor allele.

Click here to Download Table S1

Table S2. 395 ZmbHLH16-coexpressed genes of ZmbHLH16 with PCC>0.6.

Click here to Download Table S2

Table S3. Significant GO terms.

Click here to Download Table S3

Table S4. Information of 78 maize inbred lines.

Click here to Download Table S4

Table S5. Informations for recombinant vectors. In primers sequence, black letters for vector sequence, green letters for restriction sites, red letters for gene sequence, bold letters for initiation or stop codon.

Click here to Download Table S5

Table S6. ZmbHLH16 coexpressed genes homologous to *Arabidopsis* male-sterility (MS)/reproduction (MR) genes

Query gene ID	PCC-value	Subject ID	E-value	Score	Symbol	Status	Description
GRMZM2G014651	0.9972	AT1G62940	1.65E-35	147	ACOS5	MS gene (cloned)	acyl-CoA synthetase 5 (ACOS5
GRMZM2G145179	0.9972	AT1G62940	1.65E-35	147	ACOS5	MS gene (cloned)	acyl-CoA synthetase 5 (ACOS5)
GRMZM2G108894	0.9959	AT1G02050	2.06E-26	116	LAP6	MS gene (cloned)	LESS ADHESIVE POLLEN 6 (LAP6)
GRMZM2G119265	0.9937	AT1G33430	1.90E-22	104		MR gene (GO evidence)	Galactosyltransferase family protein
GRMZM2G498736	0.9800	AT4G13640	1.57E-13	73.4	UNE16	MR gene (GO evidence)	unfertilized embryo sac 16 (UNE16)
GRMZM2G073982	0.9799	AT1G16060	9.11E-13	71.6	ADAP	MR gene (mutant evidence)	ARIA-interacting double AP2 domain protein (ADAP)
GRMZM2G380650	0.9326	AT1G02050	2.93E-66	250	LAP6	MS gene (cloned)	LESS ADHESIVE POLLEN 6 (LAP6)
GRMZM2G136353	0.9323	AT3G49670	1.87E-11	68	BAM2	MR gene (GO evidence)	BARELY ANY MERISTEM 2 (BAM2)
GRMZM2G700011	0.8913	AT5G56110	2.69E-09	59	AtMYB103	MR gene (GO evidence)	myb domain protein 103 (MYB103)
GRMZM2G177928	0.8662	AT3G59530	1.95E-06	51.8	LAP3	MR gene (GO evidence)	Calcium-dependent phosphotriesterase superfamily protein
GRMZM2G040905	0.8613	AT4G13640	1.19E-12	73.4	UNE16	MR gene (GO evidence)	unfertilized embryo sac 16 (UNE16)
GRMZM2G091822	0.8375	AT1G69500	1.37E-17	87.8	CYP704B1	MR gene (GO evidence)	cytochrome P450, family 704, subfamily B, polypeptide 1 (CYP704B1)
GRMZM2G435993	0.8225	AT4G03550	4.84E-55	214	ATGSL05	MR gene (GO evidence)	glucan synthase-like 5 (GSL05)
GRMZM2G093408	0.7987	AT3G04620	6.70E-12	68		MR gene (GO evidence)	Alba DNA/RNA-binding protein
GRMZM2G368610	0.7867	AT2G44810	2.58E-06	50	DAD1	MR gene (GO evidence)	DEFECTIVE ANTHER DEHISCENCE 1 (DAD1)
GRMZM2G055279	0.7760	AT5G59030	1.67E-11	66.2	COPT1	MR gene (GO evidence)	copper transporter 1 (COPT1)
GRMZM2G155699	0.7598	AT2G35270	6.55E-07	51.8	GIK	MR gene (GO evidence)	GIANT KILLER (GIK)
GRMZM2G027522	0.7481	AT4G24972	2.85E-08	57.2	TPD1	MR gene (GO evidence)	TAPETUM DETERMINANT 1 (TPD1)
GRMZM2G060513	0.7017	AT3G04620	2.61E-14	77		MR gene (GO evidence)	Alba DNA/RNA-binding protein
GRMZM2G161680	0.6947	AT1G54830	6.31E-45	179	NF-YC3	MR gene (mutant evidence)	nuclear factor Y, subunit C3 (NF-YC3)
GRMZM2G166855	0.6895	AT4G21150	3.32E-20	98.7	HAP6	MS gene (cloned)	HAPLESS 6 (HAP6)
GRMZM2G007146	0.6734	AT4G28395	4.05E-06	51.8	ATA7	MR gene (GO evidence)	ANTHER 7 (A7)
GRMZM2G083111	0.6656	AT1G25540	8.61E-06	48.2	PFT1	MS gene (cloned)	PHYTOCHROME AND FLOWERING TIME 1 (PFT1)

Biology Open • Supplementary information

Query gene ID	PCC-value	Subject ID	E-value	Score	Symbol	Status	Description
GRMZM2G113959	0.6534	AT4G13890	8.50E-06	48.2	EDA36	MR gene (GO evidence)	EMBRYO SAC DEVELOPMENT ARREST 37 (EDA36)
GRMZM2G168228	0.6487	AT1G64110	3.25E-16	84.2		MR gene (GO evidence)	P-loop containing nucleoside triphosphate hydrolases superfamily protein
GRMZM2G123776	0.6425	AT1G60420	5.19E-25	113		MR gene (GO evidence)	DC1 domain-containing protein
GRMZM2G045287	0.6314	AT3G02230	1.32E-108	390	ATRGP1	MS gene (cloned)	reversibly glycosylated polypeptide 1 (RGP1)
GRMZM2G056039	0.6300	AT1G09080	3.38E-47	187	BIP3	MR gene (GO evidence)	BIP3
GRMZM2G062154	0.6254	AT1G53320	6.62E-38	156	AtTLP7	MR gene (mutant evidence)	tubby like protein 7 (TLP7)
GRMZM2G317051	0.6200	AT1G65470	1.96E-09	62.6	FAS1	MS gene (cloned)	FASCIATA 1 (FAS1)
GRMZM5G857992	0.6133	AT5G22420	8.33E-06	50	FAR7	MR gene (GO evidence)	fatty acid reductase 7 (FAR7)
GRMZM2G128771	0.6130	AT5G57880	1.65E-08	60.8	ATPRD2	MR gene (GO evidence)	MULTIPOLAR SPINDLE 1 (MPS1)
GRMZM2G453832	0.6123	AT4G28580	2.17E-10	64.4	AtMGT5	MS gene (cloned)	magnesium transport 5 (MGT5)
GRMZM2G036916	0.6058	AT2G35060	4.60E-21	100	KUP11	MR gene (GO evidence)	K+ uptake permease 11 (KUP11)
GRMZM2G013607	0.6044	AT4G05450	3.79E-21	100	ATMFDX1	MR gene (GO evidence)	mitochondrial ferredoxin 1 (MFDX1)
